# CON perspective

**DOI:** 10.1192/j.eurpsy.2021.217

**Published:** 2021-08-13

**Authors:** P. Falkai

**Affiliations:** Department Of Psychiatry And Psychotherapy, Univeristy of Munich, Munich, Germany

## Abstract

**Abstract Body:**

Psychiatry is facing major challenges during times of a pandemic as illustrated by the current COVID-19 pandemic. The challenges involve its actual and perceived role within the medical system, in particular how psychiatric hospitals can maintain their core mission of attending to the mentally ill while at the same time providing relief to general medicine. Although psychiatric disorders are the top leading causes of global burden of disease, we can witness mental health care being de-emphasized in the wake of the massive onslaught of the pandemic: psychiatric wards are being downsized, clinics closed, psychiatric support systems discontinued etc. in order to make room for emergency care. While nobody can deny the need to act decisively and swiftly and ramp up intensive care readiness, we believe that there is no need to do this at the expense of psychiatric care. Using the pandemic COVID-19 contingency plan developed at the Department of Psychiatry and Psychotherapy of the University Hospital of LMU Munich as a case in point, we demonstrate how a psychiatric hospital can share in the acute care of a health care system facing an acute and highly infectious pandemic like COVID-19 and at the same time provide for the mentally ill, with or without a COVID-19 infection, and develop mid and long-term plans for coping with the aftermath of the pandemic.

**Disclosure:**

No significant relationships.

